# Tract-Based Bayesian Multivariate Analysis of Mild Traumatic Brain Injury

**DOI:** 10.1155/2014/120182

**Published:** 2014-03-10

**Authors:** Yongkang Liu, Tianyao Wang, Xiao Chen, Jianhua Zhang, Guoxing Zhou, Zhongqiu Wang, Rong Chen

**Affiliations:** ^1^Department of Radiology, Affiliated Hospital of Nanjing University of Traditional Chinese Medicine, 155 Hanzhong Road, Nanjing, Jiangsu 210029, China; ^2^Department of Radiology, East Hospital, Tongji University School of Medicine, Shanghai 200120, China; ^3^Department of Diagnostic Radiology and Nuclear Medicine, University of Maryland School of Medicine, Baltimore, MD 21201, USA

## Abstract

*Purpose*. Detecting brain regions characterizing mild traumatic brain injury (mTBI) by combining Tract-Based Spatial Statistics (TBSS) and Graphical-model-based Multivariate Analysis (GAMMA). *Materials and Methods*. This study included 39 mTBI patients and 28 normal controls. Local research ethics committee approved this prospective study. Diffusion-tensor imaging was performed in mTBI patients within 7 days of injury. Skeletonized fractional anisotropy (FA) maps were generated by using TBSS. Brain regions characterizing mTBI were detected by GAMMA. *Results*. Two clusters of lower frontal white matter FA were present in mTBI patients. We constructed classifiers based on FA values in these two clusters to differentiate mTBI and controls. The mean accuracy, sensitivity, and specificity, across five different classifiers, were 0.80, 0.94, and 0.61, respectively. *Conclusions*. Combining TBSS and GAMMA can detect neuroimaging biomarkers characterizing mTBI.

## 1. Introduction

More than 1.125 million people experience a mild traumatic brain injury (mTBI) each year in the United States [1]. 7-8% of mTBI patients suffer from chronic symptoms [2]. In a one-year follow-up study, Van der Naalt et al. found that 84% still displayed mTBI symptoms including headaches, irritability, memory problems, poor concentration, and fatigue [3].

Computed tomography (CT) and conventional magnetic resonance (MR) imaging results of mTBI are typically normal. Diffusion-tensor imaging (DTI) examines the molecular diffusion of water and can measure white matter microstructural integrity noninvasively. Water diffuses more readily along the direction of axonal fibers. The diffusion profile in each voxel can be measured by DTI. One of the most commonly used DTI-based feature maps is the Fractional Anisotropy (FA) map, which describes the degree of directionality of diffusion. In mTBI, DTI has been used to identify microstructural changes that cannot be detected by CT or conventional MR [4].

Tract-Based Spatial Statistics (TBSS) is an automated whole-brain analysis method which aims to address two problems in voxel-based analysis of DTI data, the alignment and smoothing issue. TBSS projects a subject's FA map to a common space, creates the mean FA image and its skeleton, and projects each subject's FA onto the skeleton. This results in a skeletonized FA image for each subject. TBSS achieves alignment between the FA skeleton and a subject's FA map without requiring perfect nonlinear registration and does not require smoothing. Therefore, TBSS could improve the sensitivity, objectivity, and interpretability of the group-level analysis of DTI data. Several studies used TBSS to examine white matter changes in mTBI [5–7].

Graphical-model-based Multivariate Analysis (GAMMA) [8, 9] is a group-level analysis method to detect linear/nonlinear interactions among brain regions and a clinical variable. Let *C* denote the clinical variable. *C* could be a group membership variable which represents presence or absence of a disease, or a demographic variable. The input to GAMMA is image-derived feature maps which are defined in the same stereotaxic space and contains potential biomarkers of *C*. GAMMA detects a set of brain regions which are jointly predictive of *C*. GAMMA is fully automatic and does not rely on any assumption about the structure form of such interactions. It has been used in brain morphometry [10], functional MR data analysis [11, 12], and lesion-deficit analysis [13].

Combining TBSS and GAMMA, we are able to detect interactions among brain regions and the clinical variable in the FA skeleton space. We propose a novel analytic method, which combines TBSS and GAMMA, for the detection of brain regions characterizing mTBI.

## 2. Methods

### 2.1. Subjects

Local research ethics committee approved this prospective study. 39 mTBI patients and 28 normal controls were recruited from the emergency department in Shanghai Dongfang Hospital, Shanghai, China, between February 2013 and August 2013.

The diagnosis of mTBI was established by using the criteria of the American Congress of Rehabilitative Medicine for mild brain injury [14]. The exclusion criteria were (1) history of significant ear surgery, (2) penetrating head injury, (3) pregnancy, (4) history of dementia or mental disorder, (5) uremia, liver cirrhosis, heart failure, pulmonary edema, coagulopathy, and renal dysfunction, (6) ischemic and hemorrhagic stroke, (7) in vivo magnetic implants (such as iron, or with cochlear implants, vascular clips, etc.) or with pacemaker, and (8) the patient being either dead or having already received cardiopulmonary resuscitation before arrival at hospital.

The control group included healthy subjects who had no neurological or psychiatric illness and no prior TBI.

### 2.2. Data Acquisition and Imaging Parameters

All MR images were acquired with a Philips Achieva 3.0T TX MRI scanner (Royal Philips, Amsterdam, Netherlands). Diffusion-tensor images were acquired with a single-shot echo-planar sequence (TR/TE = 9,000 ms/90 ms, slice thickness = 2 mm, voxel size = 2 mm ∗ 2 mm, and field of view = 256 ∗ 256 mm). Diffusion gradients were set in 32 noncollinear directions by using two *b* values (*b* = 0 and 1,000 s/mm^2^). Diffusion-tensor imaging was performed in mTBI patients within 7 days of injury.

### 2.3. DTI Data Processing

The diffusion-weighted images were preprocessed by using FMRIB Software Library [15]. The diffusion-weighted data were registered to the b0 image using an affine registration algorithm in order to minimize distortion due to motion and eddy currents. Brain Extraction Tool [16] was used to remove nonbrain tissues in the T1- and diffusion-weighted data. Skull-stripped images were visually inspected and, if necessary, manually corrected for skull-stripping error. FA images were generated by using the Diffusion Toolbox [15].

The procedure to generate skeletonized FA images was as follows. First, all FA maps were normalized to the widely used FMRIB58 FA template using the nonlinear registration algorithm in FSL [15]. Then the mean of all FA maps was created by averaging normalized FA maps. The mean FA map was the input to the tract skeleton generation step, which aims to represent all tracts common to all subjects. The skeleton of a tract is a single line (or surface) running down the center of the tract. The FA skeleton was thresholded with FA > 0.2 to exclude voxels which are primarily gray-matter or cerebrospinal fluid. The last step was to project individual subject's FA onto the skeleton. At each point of a skeleton, the maximum FA value in the perpendicular tract direction was the value of this point.

### 2.4. Graphical-Model-Based Multivariate Analysis

GAMMA is a machine learning method for biomarker detection. It is based on two principles of brain functional organization: functional segregation and integration. GAMMA models the associations among a set of brain regions and a clinical variable *C* as a Bayesian network. In this study, the clinical variable represents whether a participant has mTBI (*C* = 1) or is a normal control (*C* = 0). GAMMA is a voxel-based method. There are two main tasks in GAMMA: voxel-space partition and Markov blanket detection. In voxel-space partition, GAMMA groups voxels into functional equivalent regions. A Markov blanket of *C* is variables which are jointly most predictive of *C*. Given the Markov blanket of *C*, knowing the states of other variables provides no additional information about *C*. Therefore, variables in the Markov blanket of *C* are biomarkers of *C*. GAMMA uses a specific type of Bayesian network called Bayesian network with inverse-tree structure. The output of GAMMA is a label field which defines a set of brain regions and a Bayesian network which describes the associations among these brain regions and the clinical variable. Each region of interest (ROI) in the label field can be represented by a single variable which represents the regional state. Then we can predict *C* using these regional states.

The input to GAMMA is the skeletonized FA maps. These skeletonized FA maps are defined in the Montreal Neurological Institute (MNI) space. For each skeletonized FA map, we aimed to generate a binary effect map, in which voxels with value 1 represent FA reduction, and voxels with value 0 represent no FA reduction. The procedures to generate the binary effect map were as follows. First, we calculated voxelwise 40th percentile values, based on the skeletonized FA maps of all subjects in the normal control group, and generated a threshold map **T**. Second, we generated a binary effect map for each subject. Let *V*_*i*_(*j*) and *T*_*i*_ denote the signal intensity of voxel *i* in the skeletonized FA map *j* and the threshold map, respectively. If *V*_*i*_(*j*) < *T*_*i*_, the value of generated binary map at voxel *i* is 1; otherwise, it is 0. We used the GAMMA suite v1.2 (http://www.nitrc.org/projects/gamma_suite/) to perform GAMMA analysis.

## 3. Results

This study included 67 subjects (39 mTBI and 28 normal controls). The mean ages of the mTBI and control group were 31 (standard deviation (SD) 7.4) and 33 (SD 9.4), respectively. There was no significant difference in the mean baseline age (*P* value = 0.319 based on two-sample *t*-test). The number of female participants of mTBI participants was 11 (total number of subjects in the group = 39) and that of normal controls was 13 (total number of subjects in the group = 28). There was no significant difference in the proportion of female participants (*P* value = 0.125).

**Figure 1:**
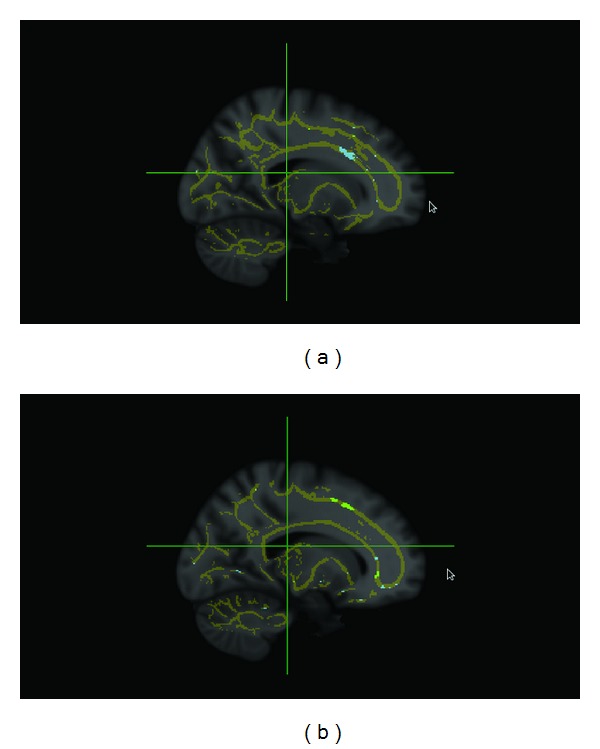
Voxels characterizing mTBI are shown in blue (ROI 1) and green (ROI 2). The white matter skeleton generated by TBSS is shown in yellow. ROIs are shown in the sagittal view and overlaid on the MNI152 template. (a) MNI coordinates *X* = 18; (b) MNI coordinates *X* = −16.

GAMMA detected two ROIs characterizing mTBI. These two ROIs are depicted in Figure 1. ROI 1 is centered on the right frontal lobe, and ROI 2 is centered on the left frontal lobe. Relative to normal controls, participants in the mTBI group demonstrated reduced FA values in these two ROIs.

Each ROI had a set of voxels. We used the regional state inference (RSI) algorithm in [17] to infer the regional states of a ROI. RSI infers the regional state using a latent-variable model, with an online Gibbs sampling algorithm. The regional state of a ROI is a biomarker characterizing mTBI. Then we constructed predictive models to differentiate mTBI and controls based on two biomarkers, regional states of ROI 1 and 2. We constructed different kinds of classifiers [18] in order to avoid the bias associated with a specific type of classifier. Classification performance was evaluated using 10-fold cross-validation.  Table 1 lists accuracies, sensitivities, and specificities of different kinds of classifiers. We found that these two biomarkers can predict *C* with mean accuracy = 0.80, sensitivity = 0.94, and specificity = 0.61.

## 4. Conclusion and Discussion

We found that biomarkers detected by combining GAMMA and TBSS accurately differentiated mTBI patients and controls. The mean accuracy, sensitivity, and specificity, across five different classifiers, were 0.80, 0.94, and 0.61, respectively.

We found that mTBI participants have decreased FA in two ROIs mainly in the frontal lobe than that in controls. White matter injury in the frontal lobe is consistently reported in TBI studies [19]. In a TBSS study of 51 mTBI patients and 50 controls, Wada et al. reported that patients with mTBI in the chronic stage had decreased FA in the superior frontal gyrus, superior longitudinal fasciculus, insula, and fornix [6]. In [20], Kraus et al. analyzed DTI data of 20 mTBI patients and 18 controls and found decreased FA in the corticospinal tract, sagittal stratum, and superior longitudinal fasciculus for the mTBI group. Our finding also suggested that DTI is sensitive to detect white matter injury in the frontal lobe in mTBI patients.

Identifying neuroimaging biomarkers for diagnosis or prognosis is of great importance. Such neuroimaging biomarkers can be identified based on expert knowledge or machine learning algorithms. The advantage of using machine learning algorithms for biomarker detection is that it can detect biomarkers in an automated, unbiased manner. GAMMA is a Bayesian machine learning method for biomarker detection. Our results suggested that GAMMA can automatically detect biomarkers in the skeletonized FA space.

One limitation of this preliminary study is the small sample size. The generated predictive model was accurate (accuracy = 0.80, sensitivity = 0.94, and specificity = 0.61). The classification performance is evaluated using tenfold cross-validation. However, we did not validate this model using an independent test data set because this study had a limited number of subjects. In future, we plan to evaluate the predictive model generated in this study in a cohort with larger sample size. We will split the data set into training and testing data sets. This could provide more reliable estimation of the generalizability of the predictive model. The predictive model generated using data from a cohort with larger sample size may have higher specificity than the current one.

In conclusion, we found that combining TBSS and GAMMA can detect neuroimaging biomarkers characterizing mTBI.

## Figures and Tables

**Table 1:** Accuracies, sensitivities, and specificities of different kinds of classifiers.

Classifier	Accuracy	Sensitivity	Specificity
Logistic model trees [21]	82	97	61
AdaBoost [22]	77	90	61
Bagging [23]	82	97	61
Naïve BN [24]	79	92	61
Support vector machine [25]	79	92	61

Mean	80	94	61
